# Effects of *Rhizobium leguminosarum* Thy2 on the Growth and Tolerance to Cadmium Stress of Wheat Plants

**DOI:** 10.3390/life12101675

**Published:** 2022-10-21

**Authors:** Dilara Maslennikova, Karina Nasyrova, Olga Chubukova, Ekaterina Akimova, Andrey Baymiev, Darya Blagova, Almaz Ibragimov, Oksana Lastochkina

**Affiliations:** 1 Ufa Federal Research Center, Institute of Biochemistry and Genetics, Russian Academy of Sciences, 450054 Ufa, Russia; 2 Department of Molecular Technologis, Ufa State Petroleum Technical University, 450000 Ufa, Russia

**Keywords:** *Rhizobium leguminosarum*, PGPR, *Triticum aestivum* L., cadmium stress, tolerance, ascorbate, glutathione, malondialdehyde, chlorophylls

## Abstract

Cadmium (Cd) stress is an obstacle for crop production, quality crops, and sustainable agriculture. An important role is played by the application of eco-friendly approaches to improve plant growth and stress tolerance. In the current study, a pre-sowing seed treatment with *Rhizobium leguminosarum* strains, isolated from the leguminous plants *Phaseolus vulgaris* (strain Pvu5), *Vicia sylvatica* (strain VSy12), *Trifolium hybridium* (strain Thy2), and *T. pratense* (strain TPr4), demonstrated different effects on wheat (*Triticum aestivum* L.) plant growth under normal conditions. Among all tested strains, Thy2 significantly increased seed germination, seedling length, fresh and dry biomass, and leaf chlorophyll (Chl) content. Further analysis showed that Thy2 was capable of producing indole-3-acetic acid and siderophores and fixing nitrogen. Under Cd stress, Thy2 reduced the negative effect of Cd on wheat growth and photosynthesis and had a protective effect on the antioxidant system. This was expressed in the additional accumulation of glutathione and proline and the activation of glutathione reductase. In addition, Thy2 led to a significant reduction in oxidative stress, which was evidenced by the data on the stabilization of the ascorbate content and the activity of ascorbate peroxidase. In addition, Thy2 markedly reduced Cd-induced membrane lipid peroxidation and electrolyte leakage in the plants. Thus, the findings demonstrated the ability of the *R. leguminosarum* strain Thy2, isolated from *T. hybridium* nodules, to exert a growth-promoting and anti-stress effect on wheat plants. These results suggest that the Thy2 strain may enhance wheat plant growth by mitigating Cd stress, particularly through improving photosynthesis and antioxidant capacity and reducing the severity of oxidative damage. This may provide a basic and biological approach to use the Thy2 strain as a promising, eco-friendly candidate to combat Cd stress in wheat production.

## 1. Introduction

Increasing agricultural crop productivity, especially cereals such as wheat, is relevant and extremely important [1,2,3,4]. One of the guiding principles of modern agricultural production is the introduction of environmentally sound and harmless approaches that are safe for human health [1,5,6,7]. Of particular importance and interest for solving this problem is the use of microbiological approaches that are based on the use of the potential of plants and microorganisms and the biological mechanisms of interaction between the components of plant–microbial systems [1,8,9]. The use of biologicals based on plant growth-promoting rhizobacterial (PGPR) strains of the genus of *Rizobium leguminosarum*, demonstrating beneficial properties of plants, is of interest. They can be successful symbionts for various plant species [10,11,12,13,14,15]. The presence of the bacteria *R. leguminosarum* can regulate in plants such processes as the increased associative fixation of molecular nitrogen [1,15,16], additional production of physiologically active compounds, including phytohormones [8,16], and improvement of the water regime of plants [16]. In addition, rhizobacteria can participate in the dissolution of hard-to-reach phosphorus compounds [17], produce antibiotic compounds that protect roots from bacterial and fungal infections [9], and modulate stress reactions in plants, thereby increasing their resistance to adverse external factors and other influences [16,18].

The complex and positive effects of the influence of PGPR on plants is widely used in plant growing practices as a seed inoculation (bacterization) technique [4]. It should be noted that the pre-sowing inoculation of seeds with microbial preparations that accelerate the growth and development of plants is an important element of crop cultivation technology aimed at obtaining friendly seedlings of cereal crops and, as a result, increasing yields [4]. It does not require complex equipment, and it provides a stable positive effect. It can be noted that the effect of microbiopreparations on plants is being studied quite actively [4,19]. However, insufficient attention has been paid to the study of the response of cereal crops to bacterization by growth-stimulating microorganisms. The application of PGPR is a sustainable approach to improving the physiological processes of crops and overcoming abiotic stresses. The effectiveness of the use of a particular bacterial preparation is often related to a particular crop, and this does not guarantee a positive effect on another crop or variety [10,11,12,13,14,16]. The maximum effect from the use of associative strains can be obtained only based on a careful selection of strains that have a greater positive effect on a particular culture. While it is important to evaluate both the growth-stimulating and protective effects of the strain, photosynthesis, components of the system’s regulating redox metabolism, and osmoprotection play an important role in the growth and development of plants under normal growing conditions [14,20]. Under stress conditions, timely changes in the operation of these systems determine the level of adaptation of a plant to stress, and the survival rate of that plant.

In connection with the growth of technogenic production, an acute problem is the pollution of soils with heavy metal ions, in particular, cadmium (Cd) ions [6,7]. Mining, smelting, and synthetic fertilizers are the main sources of Cd toxicity. Cd inhibits plant growth. Moreover, the accumulation of Cd in wheat grains poses a health risk to humans [7]. Cd ions negatively affect the seed germination, growth, grain quality, and productivity of wheat plants. In addition, Cd ions inhibit photosynthetic processes and cause the development of oxidative stress in cells [7,21]. The literature describes many different mitigation strategies to combat Cd toxicity in wheat [14,16,20]. Due to Cd ions entering wheat plants from through soil, a number of these strategies aim to reduce Cd content and its availability to wheat roots. Inexpensive and accessible strategies are often used to achieve this, such as the application of fertilizers or biochar to the soil [3,7]. In addition, the pre-sowing treatment of seeds with various phytohormones, nanoparticles, lasers, and microwaves is used [3,4,7]. In order to increase the resistance of plants to Cd, plants are treated with microbial growth regulators [1]. The use of PGPR effectively reduces the level of the negative effects of Cd on plants [10,11,12,13,14,15]. It was found that strains of the genus of *Rhizobium leguminosarum*, i.e., the rhizospheric bacterial strains Pvu5 (isolated from *Phaseolus vulgaris*), VSy12 (*Vicia sylvatica*), Thy2 (*Trifolium hybridium*), and TPr4 (*T. pratense*), stimulated the growth of roots of cucumber seedlings [22]. At the same time, the *R. leguminosarum* strain VSy12 also stimulated the growth of the roots of tomato and amaranth seedlings, while the *R. leguminosarum* strain Pvu5 only stimulated the growth of amaranth, and none of the strains exhibited growth-promoting activity on carrot roots. These results indicate that the studied strains have growth-stimulating activities for dicot plants. These strains were obtained by the researchers in [22] and there is no information about their effect on monocot plants such as wheat. This is very surprising as wheat is an important crop all over the world. The current research aims to investigate the effects of the *R. leguminosarum* strains Pvu5, VSy12, Thy2, and TPr4 on wheat growth and their ability to alleviate Cd stress.

## 2. Materials and Methods

### 2.1. Bacterial Strains

The *Rhizobium leguminosarum* strains Pvu5, VSy12, Thy2, and TPr4 were obtained from the Collection of Symbiotic Microorganisms “Symbiont” of the Institute of Biochemistry and Genetics of the Ufa Federal Research Center of the Russian Academy of Sciences (Ufa, Russia). The root nodule rhizobia strains were earlier isolated from wild-growing leguminous plants of the Southern Urals: the *R. leguminosarum* strain Pvu5—from *Phaseolus vulgaris;* the *R. leguminosarum* strain VSy12—from *Vicia sylvatica;* the *R. leguminosarum* strain Thy2—from *Trifolium hybridium;* and the *R. leguminosarum* strain TPr4—from *T. pratense.*

The isolation of pure cultures of the bacteria from nodules of the leguminous plants was carried out by the standard method [23], with some modifications [24]. DNA from the bacteria was isolated by cell lysis in a 1% Triton X100 (Serva, Heidelberg, Germany) and 1% Chelex 100 (Bio-Rad, Hercules, CA, USA) suspension [25]. Nucleotide sequences were determined on an Applied Biosystems 3500 automatic sequencer (Applied Biosystems, Inc., Sequencer Applied Biosystems, Inc., Waltham, MA, USA) using Big Dye Terminator v. 3.1.

Fragments of the 16S rRNA gene, approximately 1500 bp, were amplified using the universal primers fD1 5′-cccgggatccaagcttaaggaggtgatccagcc-3′ and rD1 5′-ccgaattcgtcgacaacagagttgatcctggctcag-3′. Phylogenetic analysis of the studied strains was performed using the MegaBlast program based on the data from the multiple alignments of the sequenced fragments of the 16S rRNA gene [26].

### 2.2. Inoculum Preparation and Seed Treatment

Cells of the *R. leguminosarum* strains Pvu5, VSy12, Thy2, and TPr4 were cultured for 2 days in glass flasks in liquid yeast mannitol YM medium (wt. % in an aqueous solution: mannitol, 1; yeast extract, 0.04; sodium chloride (NaCl), 0.01; and magnesium sulfate (MgSO_4_), 0.01) at 28 °C and 140 rpm to a concentration of 10^8^ CFU mL^−1^. To obtain an inoculum at a concentration of 10^5^ CFU mL^−1^, the stock 10^8^ CFU mL^−1^ was diluted with sterile water and monitored at 600 nm (SmartSpecTM Plus spectrophotometer, Bio-Rad, Oceanside, CA, USA).

### 2.3. Analysis of Plant Growth-Promoting (PGP) Characteristics of the Tested Bacterial Strains

A determination of indole-3-acetic acid (IAA) production was made using the Salkowski reagent [27]. The purified and freshly grown cultures on the average slopes of Luria-Bertani (LB) were transferred into tubes containing 5 mL of LB broth supplemented by 1 mg mL^−1^ of L-tryptophan (L-TRP). It was incubated at 28 ± 1 °C for 2 days. Then, the culture was centrifuged for 5 min at 1300 rpm. The Salkowski reagent (2% ferric chloride (FeCl_3_) at 0.5 M in 35% perchloric acid (HClO_4_)) was added to the supernatant at a rate of 1:2. After 20–25 min, when the color of the supernatant containing the IAA turned red, the color absorption was measured with a spectrophotometer (BioSpec_Mini, Shimadzu, Japan) at 535 nm. The uninoculated broth served as a control. The typical curve was prepared with 1, 2, 3, 4, 5, 10, 15, and 20 mg L^−1^ IAA.

The production of siderophores was detected by the universal method described in [28] using blue agar plates containing Chromium Azurol S (CAS) dye. To prepare 100 mL of the medium, 6.5 g of liquid CAS was added to 5 mL of water and mixed with 1 mL of a solution containing 1 mM of FeCl_3_ and 10 mM of hydrochloric acid (HCl). Then, 4 mL of a solution containing 7.3 mg of hexadecyltrimethylammonium bromide (HDTMA) was added to the CAS solution. The mixture was autoclaved and then added to 100 mL of LB agar medium with a pH = 6.8. Bacteria were grown on Petri dishes with “blue agar” for 7 days. The formation of a yellow halo around the blue colony was an indication of siderophore production.

The phosphate solubilization activity was tested in a Pikovskaya medium (PVK). This medium consisted of (g L^−1^): (calcium orthophosphate (Ca_3_(P0_4_)_2)_—5, glucose—20, NaCl—0.2, magnesium sulfate (MgS0_4)_-0.1, manganese sulfate (MnS0_4_)—traces, ferrous sulfate (FeS0_4_)-traces, and agar-agar-20) [29]. The bacteria were cultivated in this environment for seven days.

Nitrogen fixation was tested using Ashby’s nitrogen-free medium [30]. The bacteria were grown at 28 °C for 3 days.

### 2.4. Plant Materials and Growth Conditions

Wheat seeds (*Triticum aestivum* L., cv. Salavat Yulaev) were provided by the Chishminsky Breeding Station UFRC RAS (Bashkortostan, Russia). The seeds were sterilized in 96% ethanol for 1 min, then washed with sterile water for 2–3 min (until the smell of alcohol disappeared). Thereafter, the seeds were immersed into solutions of the strains Pvu5, VSy12, TPr4, and Thy2 (10^5^ CFU mL^−1^) or water (control) for 30 min. The seeds (100 pieces) were grown for three days on filter paper moistened with water under illumination (200 μmoL m^−2^ s^−1^) at 22–23 °C with a long-day photoperiod (16 h light/8 h dark) to select a strain that had a significant growth-stimulating effect.

To assess the protective effect of the bacterial strain, the control and inoculated seeds were grown in Petri dishes (on filter paper moistened with water (Control) and Cd—1 mM (CH_3_COO)_2_Cd) [5] under a 16/8 h light/dark photoperiod regime (200 mmoL m^−1^ s^−1^) at 22–24 °C for three days. After germination, the 3-day old seedlings were transplanted into beakers with 10% Hoagland–Arnon solution and grown for 7 days. In stressful variants of the experiment, the plants were constantly grown on a 1 mM Cd solution prepared on the 10% Hoglanad–Arnon. Plant samples of the 7-day old seedlings (roots, shoots, or whole seedlings) were taken to assess their physiological-biochemical attributes. To determine the content of the photosynthetic pigments, chlorophyll (Chl) a and Chl b, the plants’ leaves were taken. Whole plants were used to determine the content of glutathione (GSH), ascorbic acid (AsA), and proline and the activity of glutathione reductase (GR) and ascorbate peroxidase (APX), as well as the level of lipid peroxidation (i.e., malondialdehyde (MDA) content) and electrolyte leakage (EL).

### 2.5. Assessment of Growth Parameters

The germination rate was determined by how many seeds had germinated after seven days [31]. The fresh weight (FW) of the roots and shoots were recorded on the same day. For dry weight (DW) measurements, the samples were placed in a 70 °C furnace. After 48 h, measurements (DW) were carried out [32]. Data regarding shoot and root length were measured with the help of a meter rod [31]. Each variant included 30 seedlings in three biological replicates.

### 2.6. Measurement of Photosynthetic Pigments

The fresh harvested plant leaves (0.05 g) were homogenized in 90% ethanol (10 mL) with the addition of calcium carbonate (CaCO_3_) and then filtered. The optical density of the filtered extracts was measured using a SmartSpecTM Plus spectrophotometer (Bio-Rad, Oceanside, CA, USA) at 663 nm (Chl a) and 646 nm (Chl b). The Chl a and Chl b contents were expressed as mg g^−1^ FW [33].

### 2.7. Measurement of Non-Enzymatic Antioxidants

#### 2.7.1. Glutathione (GSH) and Oxidized Glutathione (GSSG) Contents

The GSH and GSSG contents from one plant mount were determined using the spectrofluoromeric method. This method is based on obtaining an o-phthalic aldehyde fluorescent product (Sigma, Australia) in accordance with the pH of the medium. Whole plant samples (approximately 0.5 g) were homogenized in 4 mL of a mixture consisting of 0.1 M potassium phosphate buffer (pH 8.0) and 25% metaphosphoric acid (HPO_3_) solution in a ratio of 3.75:1 (by volume) as recommended in [34]. The homogenate was centrifuged for 10 min at 8000× *g*, and then the supernatant was centrifuged again for 5 min at 13,000× *g*. The quantitative assessment of the GSH and GSSG in the obtained supernatant was carried out by applying the reagents specified in [35]. To assess the GSH and GSSG contents, the kinetics of the fluorescence intensity of the formed complexes were recorded at pH 8.0 and pH 12.0, respectively, using an EnSpire Model 2300 Multilabel Microplate Reader (PerkinElmer, Boston, MA, USA) at 420 nm (excitation wavelength 350 nm). The levels of glutathione forms were expressed in μmoL mg^−1^ protein.

#### 2.7.2. Ascorbic Acid (AsA) Content

The amount of AsA was determined by the titration method [35]. The wheat samples (10 g) were crushed in a porcelain mortar, extracted with 10 mL of distilled water, agitated, and filtered through a paper filter. An amount of 20 mL of filtrate was taken into a conical flask and 1 mL of 2% hydrochloric acid (HCl), 0.5 mL of 1% potassium iodide (KI), and 2 mL of 0.5% starch were added, and then stirred. The resultant mixture was titrated with 0.001 moL L^−1^ potassium iodate (KIO_3_) until it reached a stable blue color staining. The concentration of AsA was reported as mg% FW [35].

### 2.8. Measurement of the Enzymatic Antioxidants

#### 2.8.1. Glutathione Reductase (GR) Activity

GR (EC 1.6.4.2) activity was assessed by the ability of the enzyme to catalyze the reduction of GSSG using NADPH as a reductant and measuring the formation of NADP+, as expressed in units of nmoL min^−1^ mg^−1^ protein; in the calculation, the extinction coefficient for nicotinamide adenine dinucleotide phosphate (NADPH) was equal to 6.22 mM^−1^ 1 cm^−1^ [36]. Seedlings (0.25 g) were ground in liquid nitrogen by adding 0.75 mL of 50 mM Tris-HCl buffer (pH 7.6). The homogenate was centrifuged at 22,000× *g* at 4 °C for 30 min and the supernatant was used for the enzyme assay. The reaction mixture contained 50 mM Tris-HCl buffer (pH 7.6) (1.91 mL), 0.15 mM NADPH (0.02 mL), and enzyme extract (0.05 mL). Before measurement, 1 mM GSSG (0.02 mL) was added to the experimental cuvette. The reaction was monitored by a decreased in absorbance of NADPH at 340 nm.

#### 2.8.2. Ascorbate Peroxidase (APX) Activity

The activity of ascorbate peroxidase (APX, EC 1.11.1.11) was estimated by monitoring the oxidation of the ascorbate at 290 nm. [37]. The reaction mixture (2.93 mL) consisted of 50 mM phosphate buffer (pH 7.0), 17 mM (0.03 mL) ascorbic acid (C_6_H_8_O_6_), 0.03 mL ethylenediamine tetra acetic acid (EDTA), and 0.01 mL extract. The response started with the addition of 0.03 mL of 0.06% H_2_ O_2_ and was determined in the first 100 s. The data received were expressed as μmoL ascorbate oxidized mg^−1^ protein min^−1^.

The activity of all the studied antioxidant enzymes was presented, taking into account the protein content of the sample. The total soluble protein was estimated according to the Bradford method [38]. Bovine serum albumin (BSA) served as the standard. For spectrophotometric analyses, a UNICO 2800 spectrophotometer (United Products @ Instruments, Middlesex, NJ, USA) was used.

### 2.9. Malondialdehyde (MDA) Content

To determine the MDA concentration in the wheat seedlings, samples of 0.5 g were ground in distilled water and then homogenized in 20% trichloroacetic acid (C_2_HCl_3_O_2_). The homogeneous samples were centrifuged (10,000× *g*, 10 min), and the supernatant was mixed with 0.5% thiobarbituric acid (C_4_H_4_N_2_O_2_S) prepared in 20% C_2_HCl_3_O_2_ and kept in a boiling water bath (100 °C for 30 min) and then quickly cooled. The absorbance was spectrophotometrically (SmartSpecTM Plus, Bio-Rad, Oceanside, CA, USA) measured at 532 nm and 600 nm. The MDA content was calculated using an extinction coefficient of 155 mM^−1^ cm^−1^ and expressed as nmoL g^−1^ FW.

### 2.10. Measurement of Electrolyte Leakage (EL)

The state of permeability of the cell membranes was evaluated by following the EL from plant tissues registered with the use of an OK 102/1 conductivity meter HI8733 (Hanna Instruments, Inc., Sarmeola di Rubano, Padova, Italy) by measuring the ohmic resistance of the water extracts on a constant current [35]. The plant samples (1 g) were washed with running water and cut into equally sized fragments; thereafter, they were washed with running water for 3 min, rinsed with distilled water, slightly dried, supplied with 20 mL distilled water, and incubated for 1 h at 25 °C. Then, the samples were filtered and the electroconductivity of the obtained solution was measured and expressed as μSi g^−1^ FW.

### 2.11. Proline Content

The level of proline was evaluated according to [39], with modifications [40]. Fresh plants (0.5 g) and boiling water (2.0 mL) were added to the test tubes. The test pieces were placed in a water bath and boiled over a period of 30 min. After, the tubes were removed and cooled. The extract (1 mL) was mixed with an equal volume of acid ninhydrin (C_9_H_6_O_4_) solution and glacial acetic acid (CH_3_COOH). The samples were then incubated at 100 °C for 1 h and cooled in an ice container. The optical densities of the solutions were measured at 520 nm (SmartSpecTM Plus spectrophotometer, Bio-Rad, Hercules, CA, USA). The proline contents (μmoL g^−1^ FW) were calculated using a calibration curve.

### 2.12. Statistical Analysis

All microbiological, molecular, biochemical, and physiological experiments were performed in three or more bioassays and three or four analytical tests. The arithmetic average values and confidence intervals calculated from the standard error are shown in the table and graphs (± SEM). Statistically significant differences between the mean values were evaluated using analysis of variance (ANOVA), followed by the Tukey test (*p* < 0.05).

## 3. Results

### 3.1. Effect of Rhizobacteria Strains on the Growth Parameters and Leaf Chlorophyll Content in Wheat Plants under Normal Conditions

**Figure 1:**
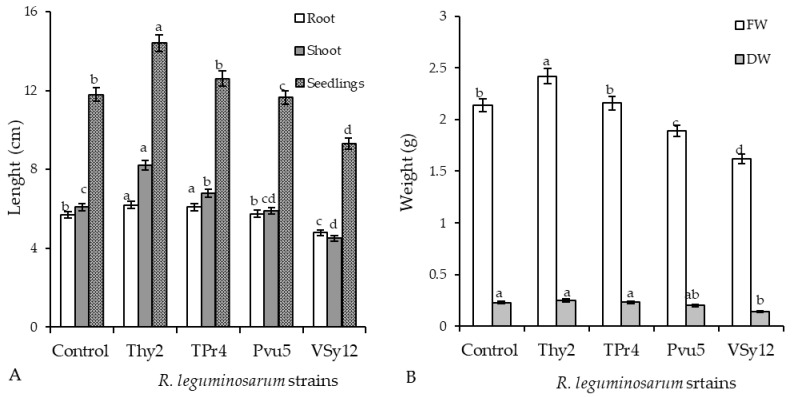
Effect of the strains of *R. leguminosarum* on the length of 7-day-old wheat seedlings: (**A**) fresh weight FW and (**B**) dry weight DW under normal growth conditions. The bars indicate the mean values of three replicates ± SEM. Different letters indicate a significant difference between the means at the level of *p* < 0.05.

Analysis was carried out on *R. leguminosarum* (strains Pvu5, VSy12, Thy2, and TPr4)-treated wheat seeds 7 days post-germination under normal conditions. It was revealed that the strain Thy2 increased the vigor of seed germination by 4% (Table 1), the length of seedlings by 120% (Figure 1A), and the FW and DW by 115%. Seed pre-treatment with TPr4 increased seed germination, the length of seedlings, and biomass, though not significantly. At the same time, the strain VSy12 had an inhibitory effect on these parameters. Taking together, these results demonstrated that wheat seeds differently responded to treatment with tested strains, showing a positive response to the Thy2 and TPr4 strains and a negative response to the VSy12 strain. For the Pvu5 strain, the studied parameters were at the control level (Table 1 and Figure 1).

**Figure 2:**
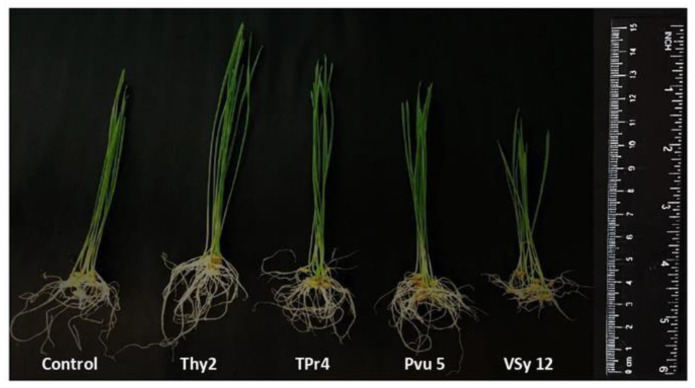
Appearance of the *T. aestivum* L. 7-day-old wheat seedlings. The seeds were inoculated with different strains of *R. leguminosarum*.

The appearance of the plants presented in Figure 2 demonstrates that the Thy2-pretreated plants looked larger compared to plants of the control and the other bacterial treatments. Plants grown from seeds pretreated with the strain TPr4 were significantly shorter than the Thy2-treated plants, but taller than the Pvu5- and VSy12-treated ones. The plant growth of the Pvu5-pretreated plants was at the level of the control, while the plants pretreated with the Vsy12 strain appeared to grow much slower than control (Figure 2).

**Figure 3:**
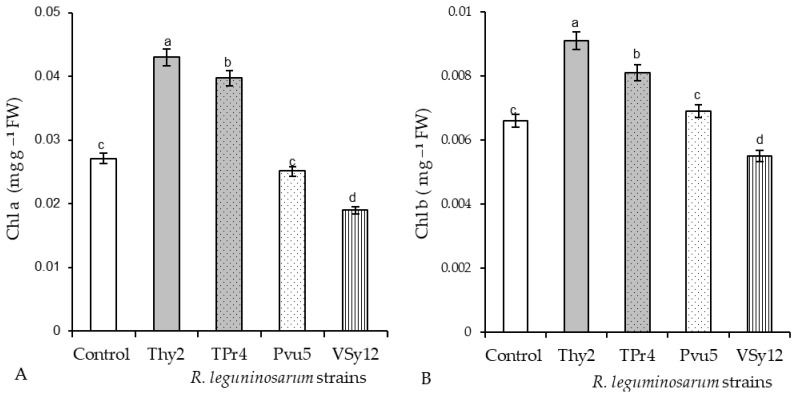
Effects of the strains of *R. leguminosarum* on the contents of Chl a (**A**) and Chl b (**B**) in the leaves of the 7-day-old wheat seedlings. The bars indicate the mean values of three replicates ± SEM. Different letters indicate a significant difference between the means at the level of *p* < 0.05.

It was revealed that in the wheat leaves pretreated with Thy2, the content of Chl a increased by 160% and Chl b by 140% above the control values (Figure 3). Seed treatment with the strain TPr4 increased the content of Chl a and Chl b, but significantly less than the strain Thy2. The content of chlorophyll in wheat leaves pretreated with Pvu5 was at the level of the control values. The strain VSy12 reduced the content of Chl a by 70% and Chl b by 83% relative to the control level (Figure 3).

### 3.2. Thy2 Strain Exerts the Main PGP Traits

**Figure 4:**
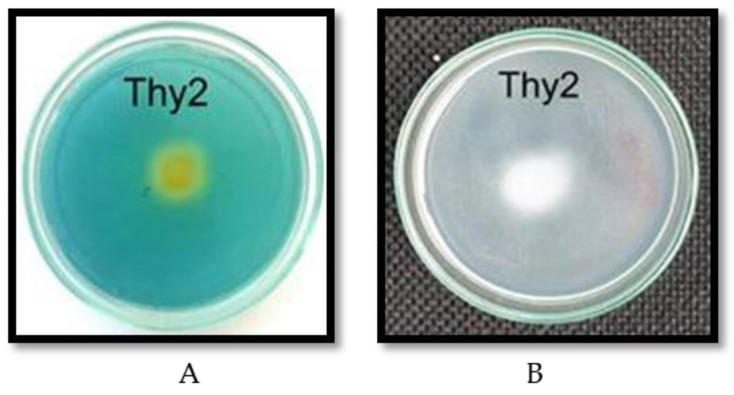
Siderophore production (**A**) and nitrogen fixation (**B**) activity of the Thy2 strain.

The results showed that the Thy2 strain had the ability to produce siderophores (Table 2, Figure 4A) and IAA and was capable of nitrogen fixation (Figure 4B). The ability to solubilize phosphate was not detected in the tested strain (Table 2).

### 3.3. Evaluation of the Protective Effect of the Thy2 Strain on the Growth and Physio-Biochemical Parameters of Wheat Plants

#### 3.3.1. Effect of Thy2 Strain on Wheat Growth under Cd Stress

**Figure 5:**
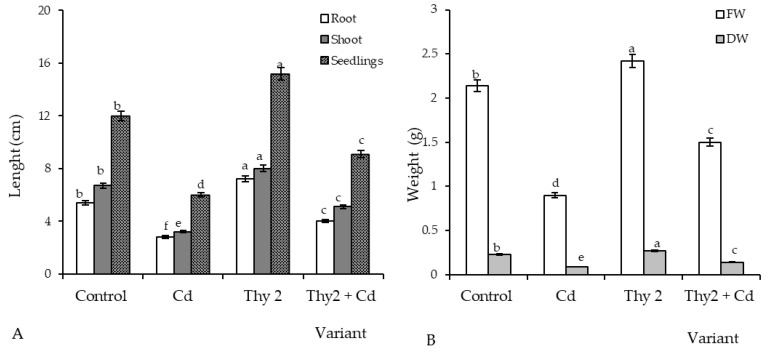
Influence of the presence of cadmium (Cd) on the length (**A**) and weight (**B**) of the uninoculated and inoculated (with *R. leguminosarum* Thy2) 7-day-old wheat plants. The bars indicate the mean values of three replicates ± SEM. Different letters indicate a significant difference between the means at the level of *p* < 0.05.

**Figure 6:**
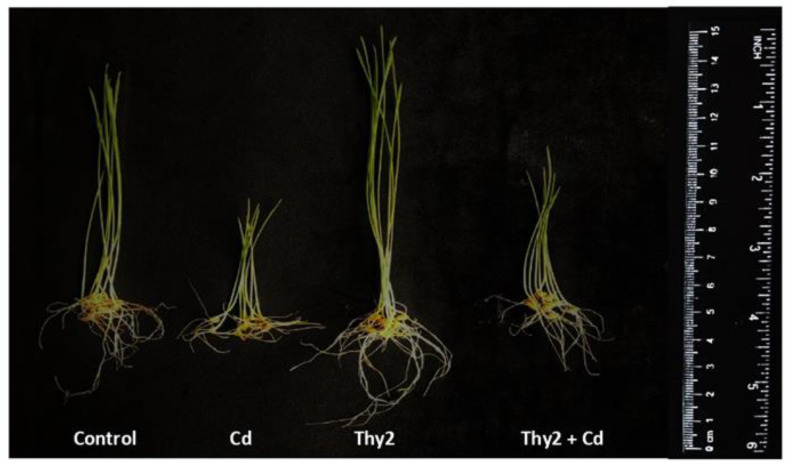
Appearance of the pretreated (with the *R. leguminosarum* strain Thy2) and untreated 7-day-old wheat plants grown under normal and Cd stress conditions.

Cd stress leads to a decrease in seed germination of 60% (Table 3). Two-fold reductions in the length and biomass (FW and DW) of the stressed wheat plants were revealed (Figure 5 and Figure 6). Wheat grown from the seeds inoculated with the Thy2 strain showed improved seed germination that was 58% higher than the uninoculated ones under the same stress conditions (Table 3). In addition, the bacterial-inoculated and Cd-stressed plants were longer and heavier than the uninoculated and the same stressed variants of the experiment by more than 1.5 times (Figure 5 and Figure 6).

#### 3.3.2. Effect of the Thy2 Strain on the Content of Chlorophyll in the Leaves of Wheat Plants under Cd Stress

**Figure 7:**
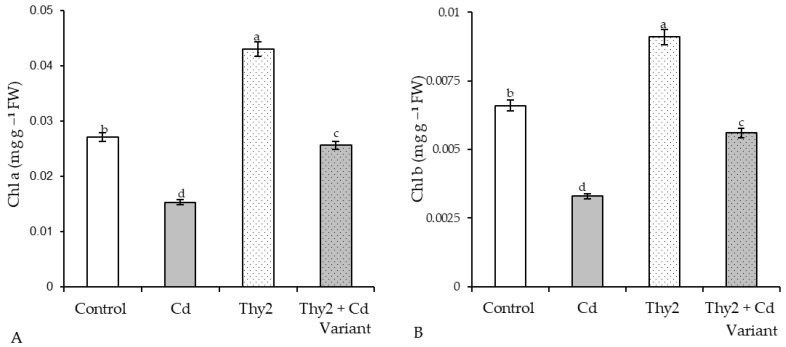
Effect of Thy2 on the content of Chl a (**A**) and Chl b (**B**) in the leaves of the 7-day-old wheat seedlings under Cd stress. The bars indicate the mean values of three replicates ± SEM. Different letters indicate a significant difference between the means at the level of *p* < 0.05.

The incubation of plants in the Cd solution led to an almost two-fold drop in the content of Chl a (Figure 7A) and Chl b (Figure 7B) in the leaves. In the plants pretreated with the Thy2 strain, the content of chlorophyll was 62–67% higher than that in the stressed plants (Figure 7). The appearance of the plants treated with Thy2, as shown in Figure 6, demonstrated that the pretreated plants appeared physiologically to be more safely stressed.

#### 3.3.3. Effect of the Thy2 Strain on the Content of Non-Enzymatic Antioxidants in the Wheat Plants under Cd Stress

**Figure 8:**
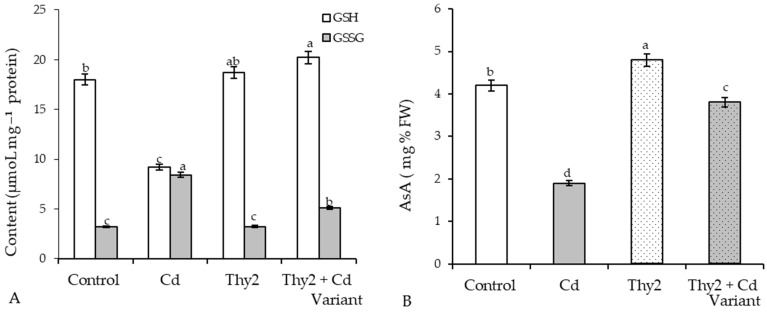
Effect of Thy2 on the content of GSH, GSSG (**A**), and AsA (**B**) in the 7-day-old wheat seedlings under normal and Cd stress conditions. The bars indicate the mean values of three replicates ± SEM. Different letters indicate a significant difference between the means at the level of *p* < 0.05.

Exposure to Cd stress led to a two-fold fall in GSH and the same level of GSSG ac- cumulation (Figure 8A). Cd stress led to a two-fold drop in ascorbic acid (AsA) content (Figure 7B). Seed treatment with the Thy2 strain resulted in a slight accumulation of GSH and AsA under normal growth conditions. At the same time, the content of GSSG remained at the level of the control values. Seed pretreatment with *R. leguminosarum* Thy2 led to an additional GSH accumulation (by 15% relative to the control) and a decrease in the level of GSSG (by 60%) (Figure 8A). The level of AsA in these plants was two times higher than that in the stressed plants, but it did not reach the control value (Figure 8B).

#### 3.3.4. Effect of Thy2 on the Activity of Enzymatic Antioxidants in the Wheat Plants under Cd Stress

**Figure 9:**
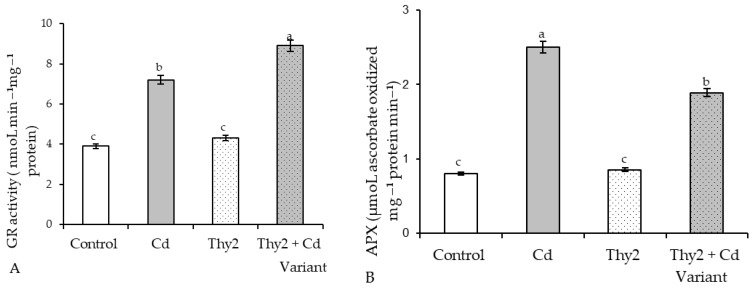
Effect of Thy2 on the activity of GR (**A**) and APX (**B**) in the 7-day-old wheat seedlings under normal and Cd stress conditions. The bars indicate the mean values of three replicates ± SEM. Different letters indicate a significant difference between the means at the level of *p* < 0.05.

Cd stress led to an increase of 1.8 and 3 times the activity of GR and APX in wheat plants, respectively. Pretreatment with the Thy2 strain did not affect the activities of enzymes, as their levels remained at the control values (Figure 9). Under stress conditions, the plants pretreated with Thy2 were characterized by additional GR activation (130%) of the stress level (Figure 9A), while the APX activity in these plants was 76% lower than its level in the stressed control (untreated) plants (Figure 9B).

#### 3.3.5. Effect of Thy2 on Proline in the Wheat Plants under Cd Stress

**Figure 10:**
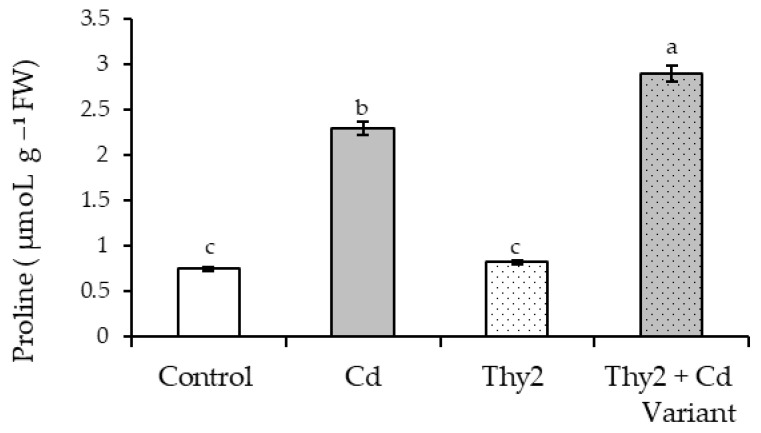
Effect of Thy2 on the proline content of the 7-day-old wheat seedlings under normal and Cd stress conditions. The bars indicate the mean values of three replicates ± SEM. Different letters indicate a significant difference between the means at the level of *p* < 0.05.

It was revealed that Cd stress resulted in a three-fold proline rise in wheat seedlings (Figure 10). However, pretreatment with the Thy2 strain reduced the concentration of stress-induced proline accumulation by 68% (Figure 10). Under normal growth conditions, pre-treatment with the Thy2 strain left the proline at the control level.

#### 3.3.6. Effect of Thy2 on the Membrane Stability in the Wheat Plants under Cd Stress

The results showed that Cd stress led to a more than two-fold increase in membrane lipid peroxidation (as judged by MDA concentration) and leakage of electrolytes (EL) from plant tissues (Table 4). However, pretreatment with the Thy2 strain significantly mitigated the stress-caused damages. Thus, the content of MDA and the leakage of electrolytes was higher (relative to the control) by 1.5 times and by 1.6 times, respectively. At the same time, treatment with the Thy2 strain itself did not affect the state of the membrane structures.

## 4. Discussion

An important indicator for assessing the prospects for the use of bacteria is the assessment of their influence on seed germination and growth parameters. During the work, it was found that the Thy2 strain stimulated the germination of seeds (Table 1) and the growth of roots and shoots (Figure 1A). It should be noted that other strains had an ambiguous effect on the growth rates of wheat. This corresponds to the literature data indicating the absence of a universal stimulating effect of bacteria on the germination and further development of plants [41]. In addition, the Thy2 strain caused a significant accumulation of both the fresh and dry biomass of wheat plants (Figure 1B). It is well known that dry biomass is a product of the photosynthetic activity of plants [2]. The Thy2 strain increased the content of chlorophylls a and b, which indicated an increased level of photo- synthetic activity in the leaves of these plants (Figure 3). Thus, based on the data obtained, it can be concluded that the Thy2 strain stimulates the growth of wheat plants, which was confirmed by the visual assessment of the tested plants (Figure 1 and Figure 2). Moreover, the Thy2 strain showed PGP characteristic such as IAA synthesis, siderophores production, and nitrogen fixation activity. The production of IAA is an essential tool for PGPR to stimulate and facilitate plant growth [42]. IAA synthesizing rhizobacteria stimulates the growth of the root system and the number of lateral roots, which leads to a better acquisition of nutrients and an increase in the development and productivity of plants [43]. Park et al. 2021 [44] obtained a bacterial strain from food waste that synthesized IAA (16.6 mg L^−1^) and demonstrated a growth-promoting effect on apple mint and chrysanthemums. Our results showed that the Thy2 strain synthesized 10 mg L^−1^ and also stimulated wheat growth (Table 2). Moreover, the Thy2 strain showed the ability to produce siderophores, which is another one of the most important signs of PGPR traits that are involved in plant growth stimulation [16,18]. Siderophores are molecules of low molecular weight (<1000 Da) with a great specificity and affinity for chelate or the link Fe3+. They play an important role in stimulating plant growth, enhancing sustainability and protecting against pathogens. [45]. Since the Thy2 strain was found to grow on a nitrogen-free medium, it has the potential for nitrogen fixation. Nitrogen is a vital element for plant growth; it is required for the synthesis of macromolecules such as amino acids, nucleic acids, and chlorophyll. According to the literature data, nitrogen fixation is one of the most important functions of *Rizobium leguminosarum* L. [1]. The obtained data proved that the Thy2 strain has PGP traits (Table 2).

In addition, the results showed that Thy2 had a protective effect on the growth of wheat seedlings exposed to Cd stress (Table 3). Inoculation with Thy2 increased germination percentage, the size of seedlings (roots, shoots), and their fresh and dry weights under Cd conditions when compared to the control (Figure 5 and Figure 6). Further, the Thy2 strain significantly reduced the Cd-caused degradation of chlorophylls a and b (Figure 7).

The application of PGPR increased the wheat growth and photosynthesis and decreased Cd uptake both in shoots and roots [1]. These studies showed that microbes can be used for the reduction of Cd toxicity in plants. Rhizobia, along with other amendments such as biochar, have enhanced the growth and yield of crops under either normal or stressful conditions [46,47].

Thereby, Thy2 has the ability to decrease the adverse effects of Cd stress in wheat plants and increase the level of photosynthetic activity. This is also evidenced by the appearance of the plants. Many studies have shown that PGPR treatment has a positive effect on plants, even in the presence of heavy metals (HMs) in the medium. For example, in the alfalfa plant *Medicago sativa* L. treated with PGPR and grown in the presence of Cu, Pb, and Zn, shoot length increased by 22–77% and shoot biomass increased up to 220% compared with untreated plants [14,20]. Treatment of the *Atriplex halimus* and *Arthrocnemum macrostachyum* plants growing in the presence of HMs led to an improvement in their morphometric parameters compared to untreated controls. This may be due to the fact that microorganisms produce siderophores, improve plant nutrition by nitrogen fixation, and boost plant growth by secreting auxins [10,12,13]. Thus, the resistance of plants to the toxic effect of Cd may be due to more efficient root growth because of the positive effects of the substances released by microorganisms.

It is well known that the antioxidant system plays a fundamental role in maintaining the redox homeostasis of plants under stress [48]. As expected, the presence of Cd in the growth medium led to the development of oxidative stress [7,48], which was accompanied by the depletion of glutathione (GSH) and ascorbate (AsA) pools, as well as the stress-induced activation of GR and APX. The overaccumulation of ROS led to the excessive synthesis of MDA and an increase in the permeability of the membrane structures. An excess of MDA, the end product of lipid peroxidation, and depletion of GSH, which is a fundamental molecule regulating mitosis [3,35], led to the inhibition of plant growth under stress. Seed treatment with the Thy2 bacteria contributed to the additional accumulation GSH (Figure 8A) and the activation of GR (Figure 9A), the key enzyme for maintaining GSH pools under stress [35]. In addition, the stabilization of AsA-APX components were observed in these plants (Figure 8B and Figure 9B), which indicates that these plants survive stress more easily. The contents of AsA and GSH can characterize the resistance and adaptive capacity of plants in response to any type of stress [48]. Thus, pretreatment with the Thy2 strain increased the adaptation of the wheat plants to the effects of the Cd ions. This was also confirmed by data on the stabilization of the state of the membrane structures of these plants.

Another indicator of the physiological state of plants is the level of accumulation of proline, which performs the function of being an osmoprotectant and an antioxidant [48,49]. Exogenous proline treatment enhanced *Brassica juncea*’s tolerance to Cd via a decrease of Cd accumulation and the reestablishment of redox homeostasis [50]. Proline is an important metabolite for plant adaptation, protection, and tolerance to Cd stress. The accumulation of proline in plants is recognized as a strategy for counteracting Cd stress by adjusting osmotic potential, stabilizing membrane structures, and reducing oxidative stress [7]. It can be assumed that a significant contribution to the realization of the resistance of wheat plants when treated with the Thy2 strain under conditions of Cd stress is the additional accumulation of proline (Figure 10). This effect of the bacteria provides a decrease in the damaging effect of Cd ions to wheat (Table 4).

## 5. Conclusions

The present study showed that seed inoculation with *R. leguminosarum* Thy2, isolated from *T. hybridium* nodules, had a growth-promoting and protective effect on wheat plants under Cd stress. Particularly, the Thy2 strain exhibits PGP properties, producing IAA, siderophores, and fixing N, and its application in a liquid formulation enhanced wheat growth and biomass compared to the control under Cd stress. Moreover, the Thy2 strain contributed to the additional accumulation of proline and glutathione and the activation of glutathione reductase in wheat plants. These led to a decrease in oxidative stress and stabilized membrane structures, measured in terms of the MDA and electrolyte leakage. Thus, *R. leguminosarum* Thy2, isolated from *T. hybridium* nodules, revealed several growth-promoting traits and induced resistance in wheat plants under Cd stress through improved photosynthesis and antioxidant capacity, and it reduced the severity of oxidative damages. Evaluation of the current study suggests that the Thy2 strain can potentially be utilized as a promising alternative and an environmentally friendly approach to facilitating wheat growth and tolerance under Cd stress. With that, further field experiments are required to evaluate its full potential for mitigating heavy metals-caused stress in plants.

## Figures and Tables

**Table 1:** Effect of the strains of *R. leguminosarum* on wheat seed germination. The presented data are the averages of three repetitions (*n* = 100).

*R. leguminosarum* Strains					
	Control	Pvu5	VSy12	Thy2	TPr4
Seed germination, %	96 ± 2	94 ± 1	80 ± 2	99 ± 1	97 ± 1

**Table 2:** Plant growth promotion activity of the Thy2 strain.

PGP Traits	Thy2
IAA (mg L^−1^)	10 ± 0.5
P solubization (mg L^−1^)	−
Siderophore production	+
Nitrogen fixation	+

**Table 3:** Effect of Cd stress on the seed germination (seven days after sowing) percentage of the uninoculated (control) and Thy2 strain-inoculated wheat plants. The presented data are the averages of three repetitions.

Variant	Seed Germination, %
Control	95 ± 1
1 mM Cd	56 ± 2
Thy2	99 ± 1
Thy2 + Cd	89 ± 3

**Table 4:** Malondialdehyde (MDA) content and electrolyte leakage (EL) in the untreated (control) and Thy2-pretreated 7-day-old wheat seedlings under Cd stress.

Variants	MDA (nmoL g^−1^ FW)	EL (μS^−1^ FW)
Control	45 ± 4.2	50 ± 5.2
Cd	101 ± 8.9	120 ± 9.9
Thy2	47 ± 4.9	52 ± 5.3
Thy + Cd	68 ± 4.9	80 ± 7.5

## Data Availability

Not applicable.
